# Improving birth experiences and provider interactions: Expert opinion on critical links in Maternity care

**DOI:** 10.18332/ejm/191742

**Published:** 2024-09-09

**Authors:** Julia Leinweber, Claire Stramrood

**Affiliations:** 1 Institute of Midwifery, Charite, Universitätsmedizin Berlin, Berlin, Germany; 2 Beval Beter - birth trauma education and training, Amsterdam, The Netherlands

**Keywords:** quality of care, obstetrics, midwifery, birth experience, birth trauma, quality of provider interaction

## Abstract

This article explores the Quality of Provider Interaction (QPI) within maternity care, spotlighting its crucial role in positive childbirth experiences. It emphasizes the need for trust-based relationships between women and their care providers, a necessity amplified by the profound neurohormonal sensitivities experienced during labor. Drawing from the ‘Optimizing the birth environment’ COST DEVOTION CA18211 Working Group, this article aims to provide insights and stimulate discussion on how to mitigate birth trauma and improve childbirth experiences. The study evolved through discussions on QPI, engagement with the group, a review of COST Action research, and conference contributions, leading to essential recommendations. From our dialogue and evaluation of existing literature, we identified four pivotal aspects critical to enhancing QPI: 1) Empathy and emotional availability, 2) Trauma-informed maternity care, 3) Integrating woman-centered individual and institutional attitudes, and 4) Empowering language use. We examine how these elements influence women's emotional and psychological well-being throughout childbirth and beyond, underscoring their critical contribution. This article proposes a framework to improve maternity care by enhancing the Quality of Provider Interaction (QPI). It offers practical recommendations for refining care protocols and language guidelines, emphasizing the importance of respectful, secure birthing environments. Adopting care models that prioritize high-quality provider interactions is crucial for the well-being of women and their families.

## INTRODUCTION

### Understanding the significance of women’s experiences in maternity care

The World Health Organization’s recommendations on Intrapartum Care for a Positive Childbirth Experience^1^ underscore the critical importance of women’s care experiences as a fundamental component of high-quality maternity care. The concept of Respectful Maternity Care (RMC)^1^ which acknowledges women’s experiential or embodied knowledge as an essential element of woman-centered care, has been central to these recommendations. During labor and birth, women exhibit an increased sensitivity to their birthing environment, which notably includes their interactions with maternity care providers^2,3^. Midwives and obstetricians through their intimate interactions with women during labor and birth are uniquely positioned to promote positive birth experiences, as the nature of these interactions plays a pivotal role in women’s psychological and emotional well-being during childbirth^3,4^. Adverse interpersonal experiences with midwives, nurses, or obstetricians may leave lasting imprints on the maternal psyche, influencing perinatal health and well-being and potentially increasing vulnerability to posttraumatic stress disorder (PTSD)^5^. Research indicates that interpersonal trauma exerts a more profound influence on enduring PTSD symptoms than trauma stemming from emergencies or natural disasters^6^. This is particularly relevant to childbirth, where trauma from interactions with care providers has been reported to be as, if not more, prevalent than non-interpersonal trauma, including obstetric interventions and emergencies^7,8^. Jolivet et al.^9^ pointed out that a notable lack of operational descriptions and definitions of the positive dimensions of RCM at the provider level may limit its practical implementation.

This methodology article aims to spotlight, from an interprofessional perspective, the critical role of the QPI in enhancing access to respectful care and positive childbirth experiences for women.

### Exploring the dynamics of women’s relationships with maternity care providers

Neurohormonal changes in the maternal brain during childbirth, indicating specific mechanisms that alter states of consciousness, render women particularly susceptible to environmental influences during labor and birth^2,10^. This sensitivity notably extends to interactions with intrapartum care providers. Contemporary research shows that for women to perceive childbirth as positive, they need to feel supported, in control, safe, and respected^11^. The heightened environmental sensitivity of women during childbirth underscores the importance of supportive care, which has been shown to bolster physiological labor processes and enhance women’s feelings of control and confidence^12^. Women consistently rate emotional support as the most vital form of support during labor, helping them stay focused and empowering them with resilience and courage^13^. This highlights the necessity for healthcare providers to have excellent communication skills that enable them to provide the crucial emotional support required during labor.

Emotional support is also integral to women’s perception of safety during childbirth^14^. Care providers can signal to be emotionally available to act as a secure base for women, offering a safe haven during the challenging and intense experience of childbirth^15^. Calming and supportive interactions initiated by care providers can stimulate oxytocin release, reducing fear, stress, and pain^10^. Moreover, clear, sensitive, and open communication by care providers is vital for women’s sense of control during childbirth^16^.

Midwives and obstetricians recognize that care delivered in a relational context with the woman, fosters positive interactions which are essential for negotiating optimal intrapartum care^17^. For midwives, the concept of ‘being with’ a woman encapsulates the commitment to addressing the woman’s physical and emotional needs during labor and birth^17^. Maternity care professionals themselves benefit from establishing positive relationships that foster meaningful interactions with women in their care^18^. Obstetricians and midwives experience increased job satisfaction and occupational well-being when they are able to form significant relationships with childbearing women^17^, underscoring that high-quality relationships and interactions are central to the professional identity of maternity care providers. Challenges such as high woman–provider ratios due to staff shortages and increased workloads can lead to a task-centric rather than woman-centric approach in providing maternity care, potentially hindering the achievement of high-quality interactions^19^.

### Conceptualizing Quality of Provider Interaction (QPI) in maternity care

The term ‘Quality of Provider Interaction (QPI) pertains to a woman’s perception of her care provider’s interpersonal verbal and non-verbal behaviors^20^. Interaction in this context is broadly conceptualized as a cognitive and action-based process encompassing physical acts and verbal or non-verbal communication^19^. Sorenson’s scale ranges from ‘disaffirmation’ to ‘affirmation’, with ‘affirmation’ representing interactions that acknowledge and support a woman’s individuality and personhood. In contrast, ‘disaffirmation’ denotes interactions of lower quality, where a woman’s personhood is disregarded, and she is treated as an object^20^. Research shows that QPI has a significant influence on how women feel during labor and birth^4,5,21^, which has a major effect on their overall childbirth experience^22^ and emphasizing the importance of QPI in understanding the full scope of childbirth. Continuity of care models, which enable and emphasize relational aspects of care, are more likely to promote high QPI^23^. The concept of QPI underscores the importance of women’s perceptions during birth and is thus crucial to a woman-centered approach to understanding birth experiences^24^.

High quality provider interactions, as perceived by women, often result in psychological safety during labor and birth^4^. Women have expressed the profound impact of positive interactions with maternity care providers using emotive language, describing these experiences as deeply caring, supportive, and even healing^25^. Interactions with maternity care providers that embody support and respect and that foster feelings of control and safety can lead to joy, confidence, and long-term positive psychosocial effects. These elements are integral to positive childbirth experiences^11^.

Conversely, low-quality provider interactions, marked by a lack or total absence of caring, personalized, and humanizing interactions, heighten the risk of traumatic childbirth experiences^5,21^. Traumatic childbirth is defined as an overwhelming and distressing experience with short- and/or long-term negative impacts on a woman’s health and well-being^24^. Women who feel unsupported or abandoned, particularly those with histories of sexual abuse, are more susceptible to perceiving childbirth as traumatic^26^. Negative or traumatic birth experiences often correlate with perceptions of coercive and disrespectful care environments^3^.

Women describe negative interactions with maternity care providers as leaving them feeling disempowered, desolated, suppressed, violated, and disregarded in their human rights^27^. Such low-quality interactions with providers may exacerbate the effects of already existing mental health conditions, potentially leading to postpartum depression^28^ and birth-related posttraumatic stress disorder (BR-PTSD)^5^. Achieving high-quality interactions in maternity care presents a challenge; although certain individual and pre-existing characteristics may make it easier to create a positive relationship, it is not an innate skill but rather necessitates training, evaluation, and continuous improvement^20^.

This article is a collaborative synthesis of insights from two members of the ‘Optimizing the birth environment’ COST Working Group, part of the broader COST Action DEVOTION CA18211 (2019–2023) pan-European research network aimed at addressing birth trauma and enhancing childbirth experiences on an international level^29^. This effort taps into the research expertise in birth trauma of both authors, their complementary clinical experience in midwifery and obstetrics, and their involvement in education and policy-making. Our discourse has centered on the imperative for synergistic and respectful interprofessional dynamics within maternity care teams, emphasizing woman-centered communication as fundamental.

The development of this expert opinion piece was propelled by a sequence of targeted discussions, emphasizing the significance of QPI in maternity care. These discussions were enriched by in-depth engagements with our working group, a critical review of scholarly work produced across the COST Action’s spectrum of working groups, and active contributions to COST Action conferences. This interprofessional exchange was vital for distilling the insights and recommendations presented herein.

The current article further articulates strategies to promote, develop, and sustain these key facets within global maternity care practices, tailored to the midwifery and obstetric academic audience. Merging our research with the collective expertise garnered through our COST network engagement, we endeavor to provide a nuanced perspective aimed at enhancing birth experiences and reducing trauma through improved provider interactions. This work seeks to advance midwifery and obstetrics globally, advocating for maternity care practices rooted in empathy, respect, and empowerment.

## METHODOLOGICAL APPROACH

From our dialogue and evaluation of existing literature, we identified four pivotal aspects critical to enhancing QPI:

1. Empathy and emotional availability;
2. Trauma-informed maternity care;
3. Integrating woman-centered individual and institutional attitudes; and
4. Empowering language use.

### Empathy and emotional availability

Empathy from providers is fundamental in establishing a trusting therapeutic relationship and is a critical component of the QPI^30^. Within the context of intrapartum care, empathy encompasses: 1) understanding the woman’s perspective and feelings, 2) communicating this understanding, and 3) acting upon this understanding in a therapeutic manner^31^.

Recent midwifery and obstetric literature has placed increasing emphasis on the affective competencies of intrapartum carers, including empathic communication with childbearing women^32,33^. In a study among over 2000 women with traumatic delivery experiences, they reported communication, listening to them, and supporting them better/more emotionally and practically as the top-three things caregivers could have done to prevent their traumatic experience^7^.

The need for a connection between women and their intrapartum caregivers that transcends cognitive empathy, encompassing emotional responsiveness, and availability, has been advocated^15^. Burnout among maternity care providers can diminish empathetic capacities, and has been linked to high workload, exposure to traumatic events, and lack of professional autonomy^34^. The opposite has also been identified high empathy may predispose to emotional distress, particularly in high-workload and highly protocolized settings with low professional autonomy^8^. Focusing on organizational interventions rather than solely on individual actions is pivotal in mitigating burnout among maternity care professionals, as it addresses the structural and systemic factors contributing to the problem^35^.

Diminished empathy occurs when a provider sees someone not as an individual with their own emotions but as an object^36^. This perspective makes achieving high QPI impossible. Correspondingly, a lack of empathy is a critical component in the development of disrespectful and abusive maternity care practices^37^. Thus, fostering a culture of empathy and emotional attunement in maternity care professionals is imperative for the enhancement of QPI and the assurance of dignified, supportive care experiences for childbearing women.

### Trauma-informed maternity care

At the time of birth, psychological and emotional safety for women is enhanced through the provision of trauma-informed care. This approach entails organizations and individuals recognizing the high prevalence and extensive impact of trauma, understanding potential recovery pathways, identifying signs and symptoms of trauma, integrating this knowledge into policies and practices, and actively avoiding actions that could cause re-traumatization^38^.

The physiological aspects of pregnancy and childbirth, coupled with the nature of maternity care interactions, particularly those of lower quality, can potentially trigger recollections of prior traumatic experiences in childbearing women^5^. Research indicates a high prevalence of adverse childhood experiences (ACEs) among women, especially those from lower socioeconomic backgrounds, many of whom have also endured various forms of trauma^39^.

The creation of a safe environment commences with supporting women during pregnancy to prepare for childbirth and the possibility of it evoking memories of past traumas^38^. Care providers who dedicate time to discussing women’s preferences, anxieties, and birth plans foster empowerment and a positive mindset, which bolsters women’s autonomy and aids in setting realistic expectations for childbirth.

Effective communication and interactions with maternity care providers significantly enhance childbearing women’s sense of control, a crucial factor in preventing re-traumatization during maternity care for women with a history of abuse^40^.

During labor and birth, aspects such as care providers introducing themselves, speaking in calm, soft tones, knocking before entering, dimming lights, and avoiding standing behind the birthing woman contribute to a sense of her being in control and feeling safe. Additionally, empowering the woman to make choices about her immediate birthing environment (e.g. companions, music, lighting, temperature) and how she wishes to manage her contractions (e.g. birthing positions, intuitive pushing) minimizes disruptions to the natural birth process. These practices also support the subduing of the neocortex, thus facilitating the limbic system’s role in guiding the birth process^2^.

Trauma-informed maternity care is closely linked with QPI; the two concepts support and enhance each other. For maternity care to be truly trauma-informed, high-quality interactions between maternity care providers and childbearing women are essential. Similarly, effective trauma-informed practices improve these interactions. Achieving such a level of care requires training maternity care providers workers in trauma-informed principles, ensuring all interactions align with these important values.

### Integrating woman-centered individual and institutional attitudes

The attitudes of intrapartum care providers significantly impact the QPI and, consequently, shape women’s experiences of labor and birth^3^. These attitudes are not only individual but can also be entrenched within the institutional culture where care providers operate^41^. It is essential for maternity care providers to be aware that the institutional culture of their workplaces interacts with the practices and attitudes of individual care givers. Midwives have expressed they are often not able to reconcile working according to institutional requirements with their ‘with woman’ philosophy^42^. Only when realized together, woman-centered provider and women-centered institutional attitudes have the potential to mark a progression to a transformative approach in maternity care, placing the woman’s needs, preferences, and autonomy at the heart of the care process.

This approach is recognizing the woman as the central figure in her childbirth journey and highlights the importance of her subjective experience when giving birth. Woman-centered care is characterized by a commitment to listening to and valuing the woman’s voice, ensuring her active involvement in decision-making, and tailoring care to meet her unique cultural, personal, and medical needs^43^. By focusing on the woman’s individual journey, a deeper understanding and respect for the diverse experiences of childbirth is fostered, thereby enhancing the overall quality of maternity care.

Attitudes also encompass risk perception and risk aversion. This spectrum includes a variety of practices, such as efforts to facilitate vaginal birth, normalization of interventions, valuing medical knowledge and expertise vis-à-vis women’s preferences, planning care (e.g. induction, cesarean section) based on practical or logistical considerations, and support for women’s choices that diverge from standard medical advice or protocols. Attitudinal elements also encompass awareness and capability to foster a safe and respectful care environment.

Research has identified an increasing trend towards risk-averse behavior among maternity care professionals^44^ and the same could be true for pregnant women themselves. This trend includes defensive medicine, defined as deviations from optimal practice primarily to avert patient or caregiver complaints^45^. When maternity care is steeped in a risk-averse, fear-based culture, it limits both caregivers’ and women’s sense of responsibility and decision-making autonomy during childbirth. Providing maternity care in a fear-based environment can lead to an overreliance on unnecessary interventions and surveillance, shifting the focus away from the unique needs of each woman^44^.

Thus, it is essential for maternity care providers to critically evaluate both their personal approach and the institutional attitudes of their workplaces, understanding how these perspectives interact to shape perceptions of risk, care practices, and interactions with the women under their care. Such awareness is fundamental in enabling providers to engage respectfully and sensitively with women in their care, thereby enhancing the overall quality and safety of maternity care experiences.

### Empowering language use

In light of the critical role that both verbal and non-verbal communication play in determining the QPI and women’s childbirth experiences^7^, it becomes crucial for care providers to fully comprehend the power of language in healthcare settings^46^. Effective communication, as highlighted by Cox and Fritz^47^, is fundamental to meaningful interactions. The language used in midwifery and medical contexts, especially with terms like ‘high risk’, ‘allowed to’, and ‘trial of labor’, can inadvertently disempower women. The linguistic choices made by healthcare professionals not only shape their perception of childbearing women but also potentially influence how these women view themselves, affecting their sense of agency and capacity for autonomous decision-making. Equally important is non-verbal communication, such as tone, volume, and non-verbal expressions, including facial cues and body language, which can also contribute to or detract from women’s empowerment.

To ensure a high-quality QPI and a positive childbirth experience, the language adopted by midwives, nurses and obstetricians should be empowering, factual and propagate an equitable relationship between women and care givers – this can be achieved by clear, respectful, empathetic, realistic, and non-judgmental language while adhering to the principles of shared decicion making. To promote women’s well-being during childbirth, this language should be predominantly positive, with an emphasis on possibilities and options, except in situations where positivity is not feasible^48^. Inspired by previous literature^46,47,49^, Table 1 outlines a set of recommended practices (‘dos’) and behaviors to avoid (‘don’ts’) in language use. This guidance is intended to equip care providers with the tools to create a supportive and empowering environment for women, thereby enhancing their autonomy and the overall quality of the childbirth experience.

**Table 1:** Common language use do’s and don’ts when caring for women during childbirth including recommended language use (examples).

*Recommended language use*	*Don’t say*	*Do say*
**Simple common language instead of complicated medical terms**	Give you an i.v. with Oxytocin (Pitocin^®^/Syntocinon^®^)	Give you a medicine to make your contractions stronger through the canula in your arm Give you a medicine to make your contractions more effective through the i.v. catheter in your hand
	Dystocia	Difficulty giving birth Obstructed labor
	Dilation	Opening of the cervix
	Perineum	The muscle and tissue between vagina and anus
**Humanising and active instead of objectifying/ dehumanising and passive**	My woman	The woman I’m caring for/ supporting
	The breech with 6 cm	Mrs. A. whose baby is breech and is 6 cm dilated
	I did a delivery	I supported a woman giving birth/ I assisted (at) a birth/
	She was sectioned	She gave birth by c-section / she had a cesearean birth (active)
	She delivered She was delivered	She gave birth
**Explaining instead of anxiety provoking**	Doing an instrumental delivery	Helping the baby being born by using a ventouse cup or forceps
	Breaking the waters / rupturing the membranes	Releasing the waters / opening the protective bag of waters
	The baby is in distress / danger	We are unsure / worried about the baby’s heart rate
	Risk	Chance of (…), because (..)
**Factual descriptions instead of euphemisms / inaccuracies**	A sticker on the baby’s head	A coil spring attached to the baby’s scalp (scalp electrode)
	A drop of blood from the baby’s head	A small cut for scalp blood sampling
	See how you are progressing	Do a vaginal examination
	
**Positive or neutral words instead of negative formulation or blaming women**	Failed induction	Unsuccessful induction
	Failure to progress	Pause or slowdown in labor, labor plateau
	High-risk pregnancy	Medically complicated pregnancy
	She refused induction	She declined my advice (offer) for induction
	Compliance	Barriers to adherence – She needs more information about …/ I have not been able to convey to her why …
	She claims her pain is x/10	She reports pain is x/10
	Termination of pregnancy	Compassionate induction
**Equity instead of Patronising**	You have to…	My advice is to …, because …
	You need to	Options open for you to consider are A or B
	You are not allowed to…	I think it is better to not …,because …
	‘Darling’, ‘Sweety’	Mrs (her last name), or (her first name) if appropriate
**Describing instead of judging**	Women like her are always … (culture, background)	She is showing… (e.g. signs of being distressed) / I can tell she cares about … (e.g. physiology, informed consent)
	Her partner is so… (annoying, loud, demanding, weak, aggressive)	Her partner may be afraid / anxious / feeling helpless but showing different emotions on the outside
	She just wants attention	She is asking for help / she may need assistance regulating emotions / she is seeking her needs to be met
	She feels so entitled	She - is aware of her rights / she is empowered / she knows what is good for her
**Choices instead of established facts**	I have to place an episiotomy	I would like to place an episiotomy – do you consent?
	I’m going to examine you	I would like to examine you – do you consent?
	
**Encouraging instead of threatening**	If you don’t … (e.g. push harder), …	Keep doing what you do - you are doing great!
**Woman-centred instead of organisational focus**	The way we do it here is…	What would you prefer? What is your view on this?
	Can you hop into the bed?	What position would you wish to adopt for labor?

Maternity care providers must be vigilant in their use of language, as it plays a pivotal role in shaping attitudes and perceptions. It is imperative to employ language that builds trust, mitigates power imbalances, and promotes shared decision-making. The practice of informed consent and shared decision-making, alongside actively involving women in discussions about the management and interventions of labor and delivery, are crucial. Such practices enhance the autonomy of birthing women, bolstering their sense of control, which is critical for reducing the risk to experience birth trauma. Strong, empowering relationships between women and their maternity care providers, founded on sensitive, respectful, and trauma-informed interactions, enrich women’s overall birth experiences. These relationships thrive on effective communication and a collaborative approach to decision-making.

The BRAIN acronym, advocated by the International Childbirth Education Association^50^ serves as an effective tool for facilitating the provision of information, aiding shared decision-making, and ensuring informed consent. This tool is versatile, it can be used by women to ask pertinent questions about their care (Table 2), or by providers to clearly explain the benefits and drawbacks of various care options or management strategies. This approach empowers women to make informed decisions and enhances the quality of the maternity care experience.

**Table 2:** BRAIN-acronym for informed decision making

**B**	**Benefits**	What are the benefits?
**R**	**Risks**	What are the risks?
**A**	**Alternatives**	What are the alternatives?
**I**	**Intuition**	What is your intuition telling you?
**N**	**Nothing**	What happens if we do nothing? What if we don’t do it now? What if we never do it?

## DISCUSSION

We have discussed four building stones of high-quality provider interactions in maternity care: 1) Empathy and emotional availability, 2) Trauma-informed maternity care, 3) Integrating woman-centered individual and institutional attitudes, and 4) Empowering language use.

Maternity care provider training programs must prioritize these aspects as they are crucial for promoting high-quality provider interactions. It is vital to understand that interactions of lower quality have the potential to traumatize women during labor and birth. To improve the quality of these interactions, some providers may need to revise their clinical practices and develop more effective communication skills. Institutions should advocate for and value positive, respectful interactions with childbearing women and their families, fostering an environment that enables providers to concentrate on the quality of their interactions. Practically speaking, the four building stones should be structurally incorporated in maternity care provider training programs. Not just on a theoretical level (e.g. what entails respectful and trauma-informed maternity care, and why is it important), but also with hands-on training (e.g. empowering language use), group discussions (developing woman-centered attitudes), self-reflection (e.g. empathy and emotional availability) and feedback during clinical internships regarding a students’ competencies on all four domains.

Advocating for respectful maternity care and establishing a secure birthing environment depend on policy-level recognition of the significance of women’s birth experiences for the health and well-being of women and their families. This necessitates a societal appreciation of positive birth experiences and a commitment to maternity care models that promote high-quality provider interactions (QPI). Such models enable midwives and obstetricians to build and maintain trustful relationships with women in their care.

Clinical leaders require structural support to facilitate change, by implementing and monitoring policies that focus on creating environments that foster empathetic, respectful, and trauma-sensitive interactions between maternity care providers and women during labor and birth. Ignoring the need for these improvements is untenable, as the quality of maternity care is integral to the health and well-being of women and their families.

Future studies could evaluate the effects of changes on educational, clinical and policy level related to the building stones of high-quality provider interactions – both for providers and childbearing women.

## CONCLUSIONS

In the realm of maternity care, it is essential for providers to be acutely conscious of the quality of their interactions with childbearing women. These interactions are fundamental in cultivating relationships based on respect and trust. Establishing such relationships during labor and birth is crucial, as it significantly enhances the likelihood of women perceiving their birth experiences as positive and empowering, and reduces the incidence of negative or traumatic birth experiences.

## Data Availability

Data sharing is not applicable to this article as no new data were created.
